# Isolation of Human Mitotic Protein Phosphatase Complexes: Identification of a Complex between Protein Phosphatase 1 and the RNA Helicase Ddx21

**DOI:** 10.1371/journal.pone.0039510

**Published:** 2012-06-28

**Authors:** Veerle De Wever, David C. Lloyd, Isha Nasa, Mhairi Nimick, Laura Trinkle-Mulcahy, Robert Gourlay, Nick Morrice, Greg B. G. Moorhead

**Affiliations:** 1 Department of Biological Sciences, University of Calgary, Calgary, Alberta, Canada; 2 Department of Cellular and Molecular Medicine and Ottawa Institute of Systems Biology, University of Ottawa, Ottawa, Ontario, Canada; 3 Medical Research Council Protein Phosphorylation Unit, College of Life Sciences, University of Dundee, Dundee, Scotland; Institute of Enzymology of the Hungarian Academy of Science, Hungary

## Abstract

Metazoan mitosis requires remodelling of sub-cellular structures to ensure proper division of cellular and genetic material. Faults often lead to genomic instability, cell cycle arrests and disease onset. These key structural changes are under tight spatial-temporal and post-translational control, with crucial roles for reversible protein phosphorylation. The phosphoprotein phosphatases PP1 and PP2A are paramount for the timely execution of mitotic entry and exit but their interaction partners and substrates are still largely unresolved. High throughput, mass-spectrometry based studies have limited sensitivity for the detection of low-abundance and transient complexes, a typical feature of many protein phosphatase complexes. Moreover, the limited timeframe during which mitosis takes place reduces the likelihood of identifying mitotic phosphatase complexes in asynchronous cells. Hence, numerous mitotic protein phosphatase complexes still await identification. Here we present a strategy to enrich and identify serine/threonine protein phosphatase complexes at the mitotic spindle. We thus identified a nucleolar RNA helicase, Ddx21/Gu, as a novel, direct PP1 interactor. Furthermore, our results place PP1 within the toposome, a Topoisomerase II alpha (TOPOIIα) containing complex with a key role in mitotic chromatin regulation and cell cycle progression, possibly via regulated protein phosphorylation. This study provides a strategy for the identification of further mitotic PP1 partners and the unravelling of PP1 functions during mitosis.

## Introduction

Initiation, execution and successful termination of metazoan mitosis require extensive remodelling of subcellular structures, including breakdown of the nuclear envelope, nuclear pore complex and the nucleolus. Mitotic spindles must be formed, condensed chromosomes aligned and separated and ultimately the nucleolus and nucleus re-assembled [1]. Key processes such as DNA transcription and RNA splicing are generally down-regulated during mitosis, yet some nuclear pore complex proteins and splicing factors were recently found to relocate to the spindle and kinetochores during metazoan mitosis where they are essential for proper mitotic progression [2]. Suggested functions for mitotic spliceosome elements include regulation of Topoisomerase IIα and thus decatenation of sister chromatids during mitosis or influencing microtubule-to-kinetochore interaction and spindle assembly checkpoint satisfaction [2]. These observations re-open the debate on the possible roles and regulation of presumed interphase-only enzymes such as splicing factors and other nucleic acid-regulating enzymes (e.g. topoisomerases or helicases) during mitosis.

Protein phosphorylation exerts an important regulatory role during mitosis. Mitotic kinases, including the cyclin dependent kinase 1 (Cdk1) and Aurora kinases, have been studied extensively, leading to an in-depth understanding of their key roles in mitotic phosphorylation and progression [3]. Protein phosphatases, their counteracting enzymes, were only recently recognized as equally crucial regulators of metazoan mitotic progression [4], [5]. Biochemical and functional screens identified the single protein dual specificity (DUSP) phosphatases Cdc14 and Cdc25 and the serine/threonine phosphoprotein phosphatase (PPP) family members PP1, PP2A, PP4 and PP6 as strategic mitotic regulators [4], [5], [6]. PPP inhibitors, deletion of selected interaction partners, or the introduction of PPP catalytic subunit mutants induces mitotic cell cycle arrests, underscoring the crucial role for protein kinases and phosphatases in mitotic progression [7]. However, the identity of mitotic metazoan PPP complexes and their interaction partners and substrates remains largely unknown [4], [5].

The metazoan PPP family encompasses the catalytic subunits PP1, PP2A, PP2B, PP4, PP5, PP6 and PP7 [8]. With the exception of PP5 and PP7, each phosphatase forms a complex with one catalytic subunit (PP) and one or more regulatory subunits, sometimes functioning as scaffolds. PPPs are ubiquitously expressed and interact with a number of regulatory subunits in a mainly mutual exclusive manner. This enables the inherently non-specific PPPs to target phosphorylated substrates with high specificity [9], [10].

PP1 has the largest array of regulatory subunits (>100 to date) [11], most of which form their primary interaction with PP1 via a canonical “RVxF” motif that slots into a hydrophobic pocket on the surface of PP1, opposite from the catalytic cleft [12]. Metazoan PP1 is present in 3 isoforms (α, β and γ), constantly bound to regulatory or inhibitory proteins to prevent uncontrolled phosphatase activity. This leads to a panoply of binary [13], sometimes ternary PP1 complexes [14]. Several mitotic PP1 complexes were already identified, for example, PP1 aids the activation of Cdc25B/C, a key Cdk1 activator, at mitotic onset [4]. PP1 binds CENP-E, enforcing stable attachment of mitotic chromosomes to spindle microtubules [15] and later counteracts Aurora B-mediated phosphorylation events to reduce kinetochore integrity and initiate mitotic exit [16]. PP1 also interacts with Repo-man and PNUTS at mitotic exit, forming complexes involved in chromosome de-condensation [17], [18], [19].

The core PP2A complex contains a catalytic subunit, PP2Ac and a scaffold subunit (PR65/A). Both subunits exist as two isoforms (α, β) in metazoans. Substrate specificity is achieved via regulatory (B) subunits, divided into B, B’, B” and B’’’ [20]. PP2A can also interact with viral proteins [21], a Tip41-like protein (TIPRL) or alpha4 (α4/IGBP1) [22]. The latter two are labelled general PPP interactors due to their additional binding capacity for PP4 and PP6 [6]. TIPRL can even form a trimeric complex with PP2Ac and α4 [23]. Contrary to PP1, PP2A-B’ prevents Cdk1 activation until mitosis [24]. PP2A-RSA1/2 (B”) has a positive impact on mitotic spindle assembly in *C. elegans* [25]. In HeLa cells, PP2A-B’ aids in preventing untimely separation of sister chromatids, while PP2A-B55 is key in post-mitotic chromatin decondensation and membrane re-assembly [26], [27]. PP4 is presently linked with microtubule organization and/or centrosome maturation by regulating Cdk1 activity [28], [29] while PP6 recognizes and down-regulates active, mitotic Aurora A, the latter in a complex with the mitotic spindle associated protein Tpx2 [6].

Thus, some mitotic-onset and -exit PPP complexes have been identified yet others, particularly from meta- to telophase, remain largely unknown [4], [5]. Indeed, an active role for PP1 during mitosis still remains under debate [30]. Isolation and identification of mitotic PPP complexes will be essential to resolve these issues. Here we aim to identify those mitotic PPP complexes. We synchronized human cells in mitosis, enriched the mitotic spindle-associated proteome and subjected this to PPP affinity chromatography. We thus identified the RNA helicase Ddx21/Gu as a novel PP1 interactor. We could further show that Ddx21 and PP1 also form a complex in the nuclei of unsynchronized cells. Apart from this helicase, we also identified the splicing factor Prp8 and the serine/arginine kinase SRPK1 as members of the mitotic PP1 interactome. Ddx21, Prp8 and SRPK1 were previously found in a mitotic, DNA Topoisomerase IIα-containing complex, proposed to aid TOPOIIα mediated unwinding of condensed mitotic DNA [31]. Our results suggest PP1 is part of this mitotic complex, which opens up further interesting prospects on novel roles for this phosphatase during metazoan cell division.

## Results

### Phosphoprotein Phosphatases at the Mitotic Spindle Apparatus

Recent literature provides ample indications for the key role of serine/threonine phosphatases during the onset of, progression through and exit from eukaryotic mitosis [4], [5]. However, the precise nature of the complexes involved and their direct substrates remain largely unknown. Furthermore, protein phosphatase complexes at transient structures such as the mitotic spindle apparatus are restricted in time and place. Here we aimed to enrich these low-abundance, more transient phosphatase complexes by combining cell synchronizations with subcellular fractionation and affinity chromatography.

**Figure 1:**
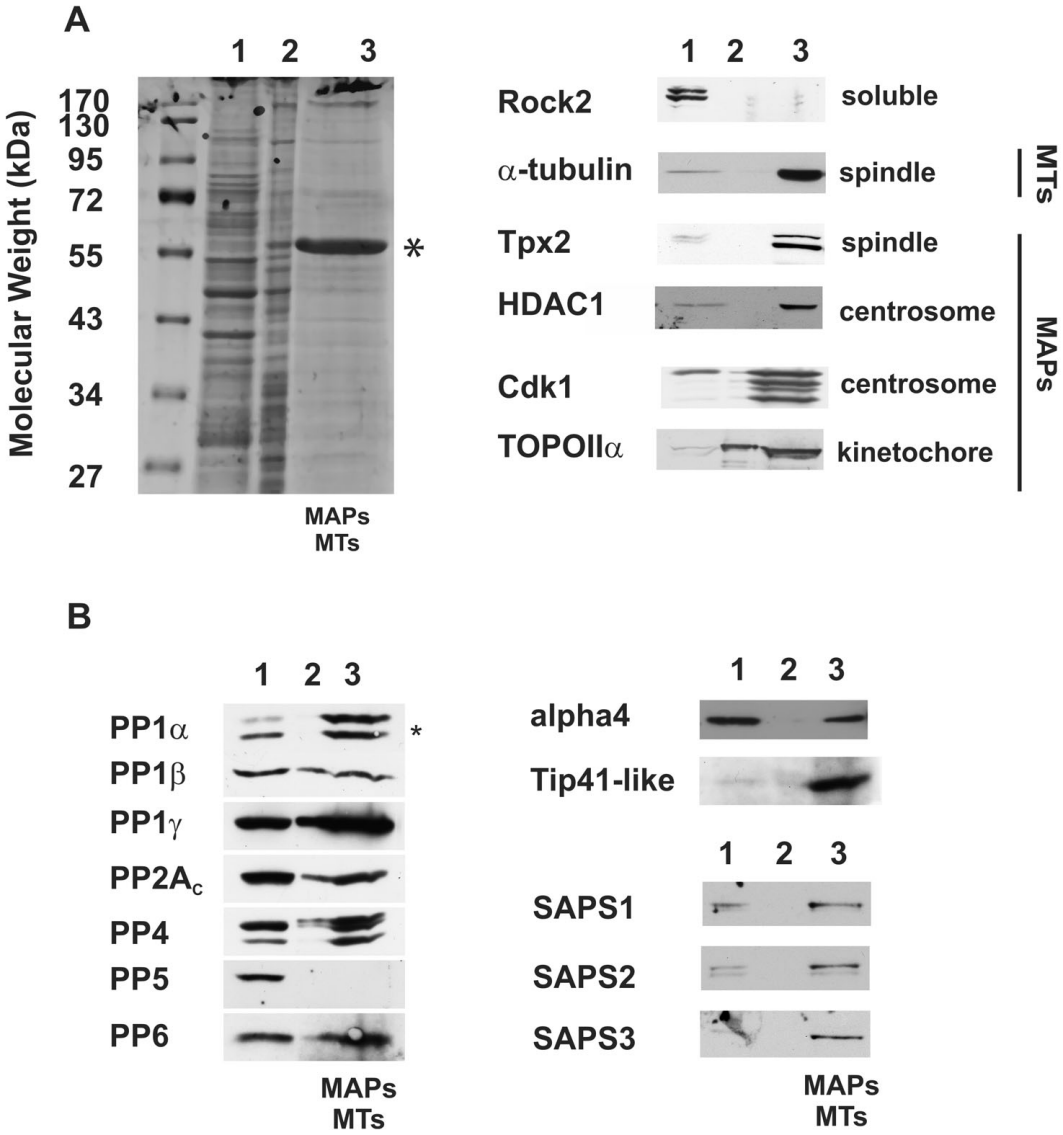
Purification of the human mitotic spindle and chromatin associated proteome and identification of PPP subunits. A. Mitotic spindle purification and associated proteome shown via colloidal stain and western blot analyses. Mitotic spindles and associated proteins were isolated according to (Fig. S1). Isolation is divided in the soluble proteins (fractions 1), low ionic strength wash (fraction 2) and mitotic spindle and associated proteome (fraction 3). Fraction 3 consists of microtubules (tubulin dimers - MTs) and Mitotic Spindle Associated (MAPs) and interacting proteins, including remnants of the microtubule organizing centre (MTOC), the spindle-associated centromere/kinetochore region and DNA-binding proteins. Proteins were separated by SDS-PAGE and visualised by colloidal stain (left), (*: enrichment of tubulin) or subjected to western blot analyses with antibodies against the indicated proteins (right). Equal protein amounts were loaded for each fraction (lane 1, 3 µg; lane 2, 3 µg), excluding the estimated total tubulin enrichment in fraction 3 (lane 3, 6.3 µg). **B.** Phosphoprotein phosphatases (PPP) in the mitotic spindle and chromatin associated proteome. Experimental set-up was according to (Fig. 1A and S1). Fractions were loaded as in A, except 20× less total protein was loaded in each lane. Western blot analyses were performed with antibodies against the indicated proteins.

Human cells (HeLa and HEK293) were synchronized with a thymidine-nocodazole block and G2/M arrested cells enriched by mitotic shake-off and released in fresh media until metaphase, as described previously [32] (Fig. S1). Cells were harvested and lysed in the presence of paclitaxel to release soluble proteins (Fig. 1 and S2, fraction 1) but maintain the mitotic spindle apparatus (Fig. S2). The actin and intermediate cytoskeleton was removed via a low ionic strength wash (fraction 2). Fraction 3 consists of the mitotic spindle associated proteins (MAPs) and microtubules (MTs), the latter made up from α- and β-tubulin dimers. This fraction also enriches the microtubule originating centre (MTOC, centrosomal proteins) and the kinetochores, proteinaceaous contact points for MTs at mitotic, condensed chromosomes [32]. Consequently, centromeric and additional DNA/chromatin associated proteins are present in fraction 3, even though we treated with DNAse/RNAse. We separated each protein fraction via SDS-PAGE and analysed them by colloidal stain (Fig. 1A left panel) or by western blot (Fig. 1A right panel) for the presence of proteins with known subcellular localizations.

The left panel of Figure 1A shows the dramatic enrichment of a ∼55 kDa protein during the isolation procedure (*). Western blot analyses confirmed α-tubulin as the major component (Fig. 1A right panel). A soluble/membrane associated protein (Rock2) remained in fraction 1 while known MAPs such as the spindle assembly factor Tpx2 or Topoisomerase IIα (TOPOIIα), a dsDNA break-and-repair enzyme that localizes at the inner centromere/kinetochore during mitosis [33], both display enhanced signal intensity in fraction 3. Histone deacetylase 1 (HDAC1) and the cyclin dependent kinase Cdk1 are partially soluble but enrich at the centrosome/mitotic spindle during mitosis [34], [35], [36]. These observations were reflected in our western blot analyses, corroborating purification of the mitotic spindle apparatus, using a previously established protocol. Although likely free of cytosolic components, we do acknowledge that this methodology does leave minor chromatin contamination. HEK293 derived spindles gave similar results to HeLa cells but at a reduced yield (data not shown).

Our aim was to isolate PPP complexes present within this subcellular proteome. We analysed the purified mitotic spindle proteome for the presence of the catalytic subunits of the PPP family enzymes via western blot analyses (Fig. 1B). Equal protein amounts were loaded on SDS-PAGE, whereby the tubulin in fraction 3 was subtracted. The band under PP1α (#) is most likely a degradation product. The 3 isoforms of PP1, PP2A, PP4 and PP6 are present in fractions 1 and 3, with higher amounts of PP1α, PP1γ and PP6 in fraction 3, compared the other phosphatase catalytic subunits in each fraction. Indeed, fraction 1 contains ∼75% of soluble cellular protein, 3 µg of which was loaded while fraction 3 represents ∼20% of the total original protein, including the majority of cellular tubulin. We loaded 6.3 µg to accommodate for the tubulin excess (see materials and methods). It is notable that PP5 is clearly present in fraction 1 but not in the mitotic spindle and chromatin interacting proteins (fraction 3). To further define the identity of the PPP complexes present in fraction 3, we probed for a small number of PP2A, PP4 and PP6 complex subunits. We examined alpha4 (IGBP1) and TIP41-like protein since they can interact with PP2A, PP4 or PP6 [6], [22], [23]. Human alpha4 gives an equally strong signal in fraction 1 and 3, suggesting it is present in soluble complexes and the spindle apparatus alike. TIP41-like protein on the other hand displays a dramatically enhanced signal in fraction 3, indicative of a potentially novel MAP and/or chromatin interacting protein. The 3 known PP6 regulatory subunits (SAPS1-3) are found in fractions 1 and 3, supporting the recently proposed key role for PP6 in mitotic progression [6].

### Microcystin-based Isolation of Mitotic Protein Phosphatases

**Figure 2:**
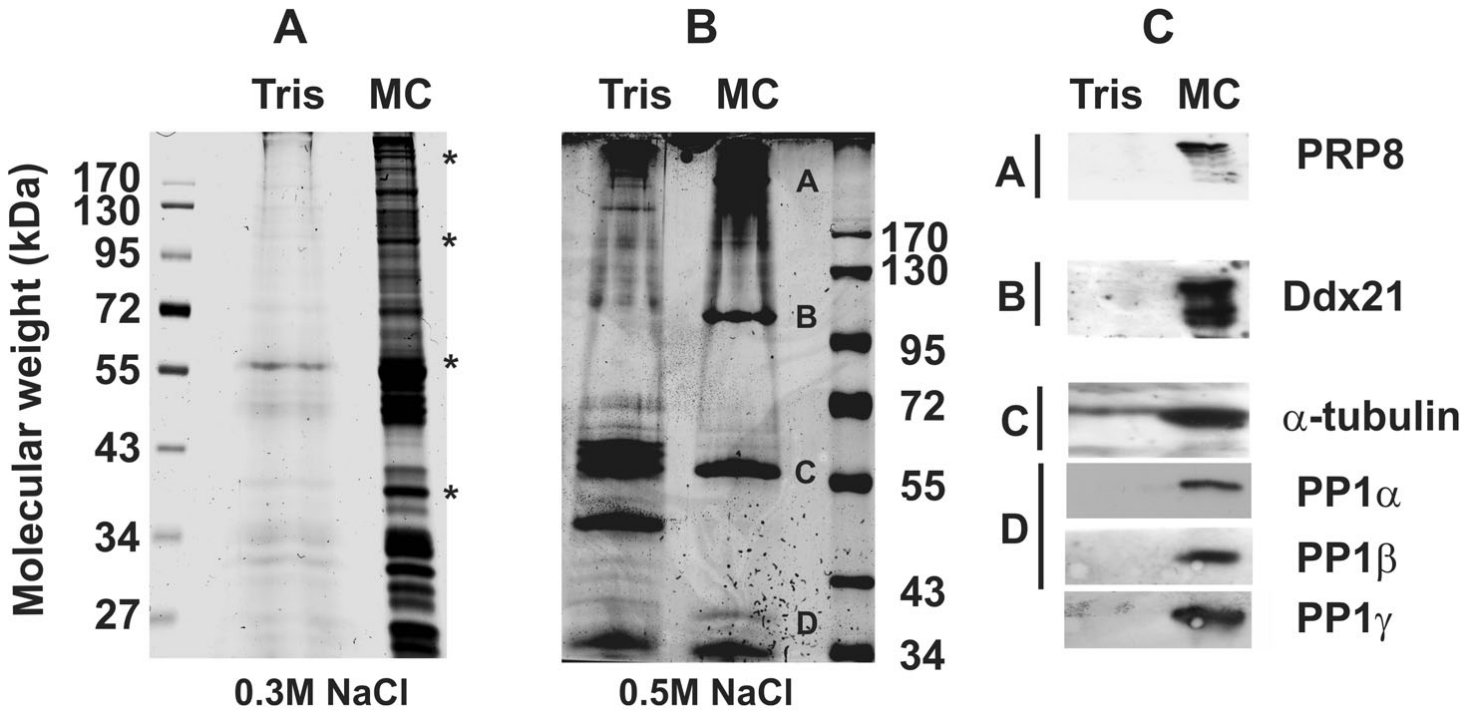
Enrichment of salt-soluble PPP complexes from the mitotic spindle proteome via MC-Sepharose affinity chromatography. The mitotic spindle and chromatin associated proteome was enriched and separated into soluble proteins and MTs according to (Fig. 1, S1–2). Soluble proteins were brought to 420 mM NaCl and PPP complexes isolated via affinity chromatography with MC-Sepharose and Tris-Sepharose as a control matrix. Matrices were washed with buffer A at low (**A**, 300 mM NaCl) or medium (**B**, 500 mM NaCl) ionic strength. Proteins were eluted with 1% SDS, concentrated, separated via SDS-PAGE and visualized by colloidal silver stain (**A**, **B**). Specific interactors (**B**, bands A–D) were isolated and identified via LC-MS/MS (Fig. S3). **C**. Western blot analyses with the respective antibodies to verify the specific presence of the interactors identified in **B**.

We showed that PP1, PP2A, PP4, and PP6 are part of the mitotic spindle and chromatin interacting proteome fraction (Fig. 1 fraction 3). We subjected fraction 3 to a buffer with enhanced ionic strength (0.6 M NaCl) in an attempt to solubilize the PPP complexes from the microtubules and any remaining chromatin. Solubilized proteins (fraction 3a – MAPs and chromatin interacting proteins) were separated from chromatin and microtubules (fraction 3b - MTs) via centrifugation (Fig. S2). Paclitaxel prevents the collapse of the mitotic spindle (Fig. S2 compare (+) and (−) lanes). The strength of the ionic interactions between MAPs and MTs hinders full solubilisation of the MAPs and chromatin interacting proteins at 0.6 M NaCl (Fig. S2 compare 3a(+) with 3b(+)). Increased ionic strength (up to 2 M NaCl or KCl) yielded more soluble MAPs (data not shown) yet these conditions may cause complex disruption. We therefore maintained our initial, moderate buffer strength (0.6 M NaCl) for further solubilisations. We enriched PPP complexes from the solubilized MAP and chromatin interacting proteome via affinity chromatography with a pan-phosphoprotein phosphatase (PPP) complex affinity matrix (microcystin (MC)-Sepharose) or control matrix (Tris-Sepharose), according to [11]. The matrix was washed with a low ionic strength buffer (0.3 M NaCl) and the full PPP interactome eluted with 1% SDS. We obtained a significant number of potential PPP interactors when compared with the control Tris-Sepharose purification (Fig. 2A). Raising the ionic strength of the wash buffer from 0.3 M to 0.5 M NaCl (Fig. 2B) reduced this number significantly to the most robust interactions. The silver stains displayed are representative of at least 3 independent experiments. The positions of the most prominent bands, labelled A–D (Fig. 2B), correspond to strongly enriched bands in the low ionic strength eluate (* Fig. 2A). We excised bands A–D for identification via mass-spectrometry (ESI-orbitrap) (sequence coverage Fig. S3). Proteins identified by the presence of numerous unique peptides were (A) the pre-mRNA processing factor (p220) Prp8; (B) the nucleolar RNA Dead Box helicase 2 Ddx21/RHII/Gu; (C) α- and β-tubulin and (D) the ribosomal protein RPL5, Fibrillarin and the phosphatase catalytic subunits PP1α and PP1β (Fig. S3). Mass spectrometry analyses did not identify peptides unique for PP1γ but its presence could be confirmed via western blot analyses using isoform specific antibodies. The other 2 PP1 isoforms (α; β) were also confirmed by western blot with isoform specific antibodies (Fig. 2C, panel D).

### The Splicing Factor Prp8 and the RNA Helicase Ddx21, Potential Novel Mitotic PP1 Interactors

Microcystin-Sepharose affinity chromatography enriches PPP complexes via the catalytic subunits of the PPP family. In this case, these complexes would be associated with mitotic spindles and/or chromosomes. Figure 2 shows that we found PP1 isoforms and a number of interacting proteins, all possible PP1 regulatory subunits and/or PP1 substrates in this fraction. Most PP1 regulatory subunits have a canonical PP1 interaction motif (RVxF) [12]. Variations of this motif are present in Prp8 namely RAVFWD (aa 1151–1156) and in Ddx21 with KGRGVTF (aa 202–208), hereafter motif 1, and RTIIF (aa 440–444) (motif 2). Tubulin, RPL5 and Fibrillarin do not possess such motifs. Nonetheless, RPL5 has already been shown to impact PP1 activity [37]. Tubulin is an abundant protein and can bind non-specifically to affinity matrices [38], yet our western blot analyses show that α-tubulin was enriched in the MC-eluates (Fig. 2C), suggesting it may be an (in)direct PP1 interaction partner. Fibrillarin is a well-known, non-specific binding partner for affinity matrices (40), and may be a contaminant here.

**Figure 3:**
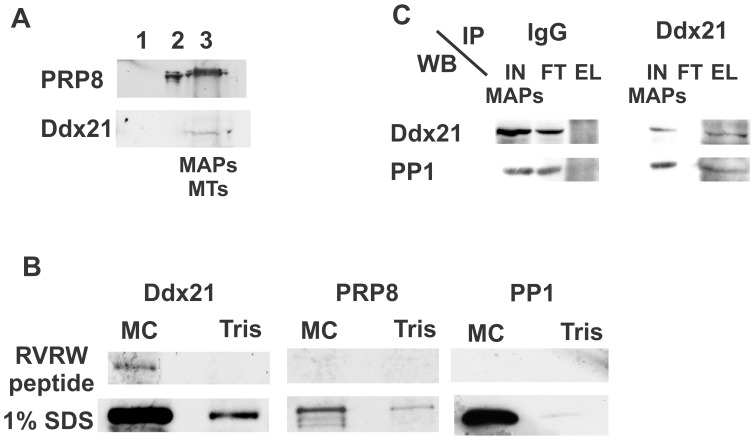
Peptide displacement partially releases mitotic Ddx21 from the MC-Sepharose matrix. A. Prp8 and Ddx21 enrich in the mitotic spindle proteome. Mitotic spindle and associated fractions were obtained and proteins loaded and separated as in Fig 1. Western blot analyses were with antibodies against Ddx21 and Prp8. **B.** A fraction of Ddx21 is displaced by an excess of a PP1-binding peptide. MAPs were incubated with MC- or Tris-Sepharose matrices and PP1-interaction partners eluted sequentially with an excess of a scrambled PP1-binding peptide (not shown) followed by a PP1-binding peptide (-RVRW-). Any remaining PPPs, including PP1, and their interaction partners were finally eluted with 1% SDS. Proteins were separated via SDS-PAGE and presence of Ddx21, Prp8 and PP1 verified by western blot analyses with the respective antibodies.

Thus, Prp8 and Ddx21 have potential PP1-binding motifs and were enriched via microcystin-Sepharose chromatography (Fig. 2C) from the mitotic spindle and chromatin associated proteome. We confirmed their presence in the mitotic spindle and chromatin associated proteome (Fig. 3A) and the microcystin-eluates by western blot analyses (Fig. 2C). Previously, we have shown that PP1-interactors, relying on their RVxF motif for binding to PP1, can be displaced from the phosphatase subunit by an excess of a peptide with an undisputed PP1 motif [11]. Here, we use this approach to attempt Ddx21 and/or Prp8 displacement. We isolated the mitotic spindle and chromatin associated proteome, solubilized associated proteins and incubated these with either MC-Sepharose or Tris-Sepharose matrices. Then, we proceeded with a sequential elution, consisting of an excess of RARA peptide (amino acids within the PP1 binding motif were changed to RARA), followed by an RVxF-motif containing peptide elution (RVRW) and finally elution with 1% SDS (Fig. 3B). Western blot analyses of the different elutions show that neither Ddx21 nor Prp8 were displaced by the RARA peptide (data not shown). The RVRW-peptide resulted in a partial elution of Ddx21, but not Prp8, while 1% SDS released the remainder of Ddx21 and all of Prp8 from the matrix. As expected [11], PP1 was present in the SDS-MC eluate only. These results suggest that the potential PP1 binding motifs in Ddx21 play at least a partial role in its interaction with PP1.

### The RNA Helicase DDx21, a Novel PP1 Interactor?

Previously, we identified Ddx21 as a member of the nuclear phosphatase proteome in HeLa cells by mass spectrometric analysis of a microcystin-enriched nuclear fraction [11]. Similar results were obtained when PP1-GFP was enriched from purified nucleoli, derived from SILAC-grown HeLa cells, using the method described in [39]. Ddx21 was recognized as a PP1-GFP interaction partner (see Fig. S4A for sequence coverage). We corroborated these nuclear interaction data with a reverse co-immunoprecipitation of the endogenous proteins, using Ddx21-antibodies as bait on a nuclear extract of growing HeLa cells. Fig S4B shows our western blot analyses, identifying PP1 as a co-eluant of Ddx21. These results define Ddx21 as a novel, low abundance PP1 interactor in interphase HeLa cells. These data further support our initial complex identification in mitotic cells.

**Figure 4:**
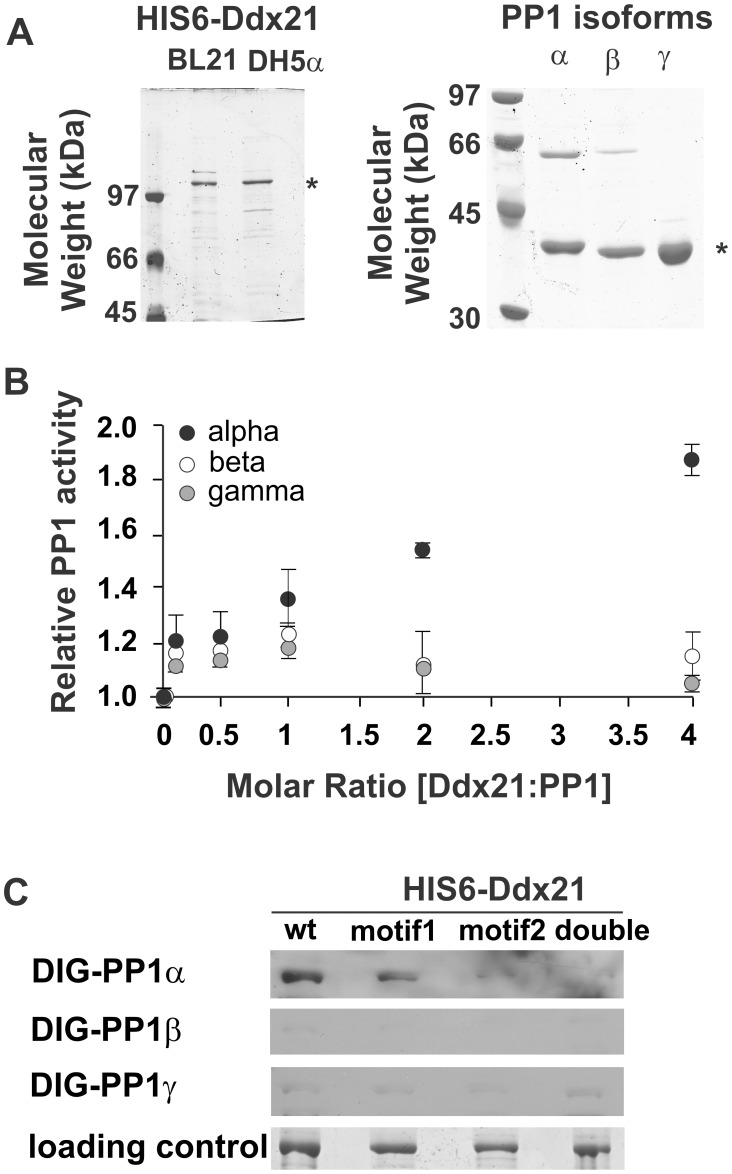
Ddx21 interacts with PP1 directly and influences PP1α activity. **A.** Bacterial expression & purification of Ddx21 and PP1. HIS6-Ddx21 was purified from DH5α or BL21-DE3* expressing cells with SP-Sepharose and Ni^2+^-NTA. PP1 isoforms were purified according to [66]. Purified Ddx21 (*) and PP1 (*) isoforms were separated via SDS-PAGE and stained with colloidal. **B.** PP1 isoform activity assay in the presence of Ddx21. Proteins were premixed according to increasing molar ratios of Ddx21:PP1 and then incubated with PP1 substrate (pNPP). Data are shown as mean ± S.D. (n = 3). PP1 activity is normalized against activity without Ddx21 (point 0 on X-axis, normalized to 1). **C.** Direct interaction between PP1 and Ddx21 occurs partially through the canonical PP1 binding motif. His6-Ddx21 (wt); His6-Ddx21_RARA_ (aa202–208: motif 1); (aa440–444: motif2) and (double) were expressed in and purified from DH5α cells. Proteins were separated via SDS-PAGE and loaded amounts verified by colloidal stain (loading control). Purified PP1α, β and γ isoforms were labelled with DIG and overlay assays were executed and visualised with α-DIG antibodies.

Next, we investigated a potentially direct interaction via binary interaction studies, i.e. Far westerns, PP1 activity assays and *in vitro* pull downs with bacterially expressed and purified Ddx21 and PP1 isoforms. We cloned and expressed wild type His6-Ddx21 (wt) and His6-Ddx21 alleles mutated in either one (motif 1, motif 2) or both (double) of the potential PP1-binding motifs. Both BL21(DE3) and DH5α bacterial strains can express His6-Ddx21 (wt) (Fig 4A), yet yield and protein stability for all 4 Ddx21 alleles is more robust in DH5α (data not shown). PP1 isoforms (Fig. 4A) were expressed and purified to near homogeneity.

We first studied the impact of the helicase presence on PP1 phosphatase activity (Fig. 4B). Since the native substrate of this complex remains to be identified, we used the small molecule substrate para-nitrophenyl phosphate (pNPP). We incubated each PP1 isoform with increasing amounts of His6-Ddx21_wt_ to a molar excess of 4∶1 (Ddx21:PP1) and measured PP1 activity towards pNPP. Interestingly, where PP1β and PP1γ activity shows no alteration in the presence of increasing amounts of Ddx21, PP1α becomes more active towards the substrate. This increasing activity plateaus at an 8–16 molar excess of Ddx21 to PP1α (data not shown).

These observations suggest PP1α and Ddx21 may interact *in* *vitro* and PP1 activity could be influenced by Ddx21 presence. We performed Far-western blot analyses to independently corroborate this interaction. We made DIG-labelled PP1 isoforms and confirmed their functionality against crude HeLa and bacterial lysates (positive control) (data not shown). Far-Western blot analyses (Fig. 4C) show that DIG-PP1α interacts with His6-Ddx21_wt_ and His6-Ddx21_motif1_ but has a severely reduced affinity for either His6-Ddx21_motif2_ or His6-Ddx21_double_. Equal amounts of all 4 Ddx21 alleles were used (loading control, Fig. 4C), suggesting that motif 2 is required for the interaction between PP1α and Ddx21. Similar experiments with DIG-PP1β, γ indicate these isoforms do not interact significantly with Ddx21 under the conditions employed during Far-Western analyses (Fig. 4C). These results support our previous observation where Ddx21 presence influences PP1α activity (Fig. 4B).

**Figure 5:**
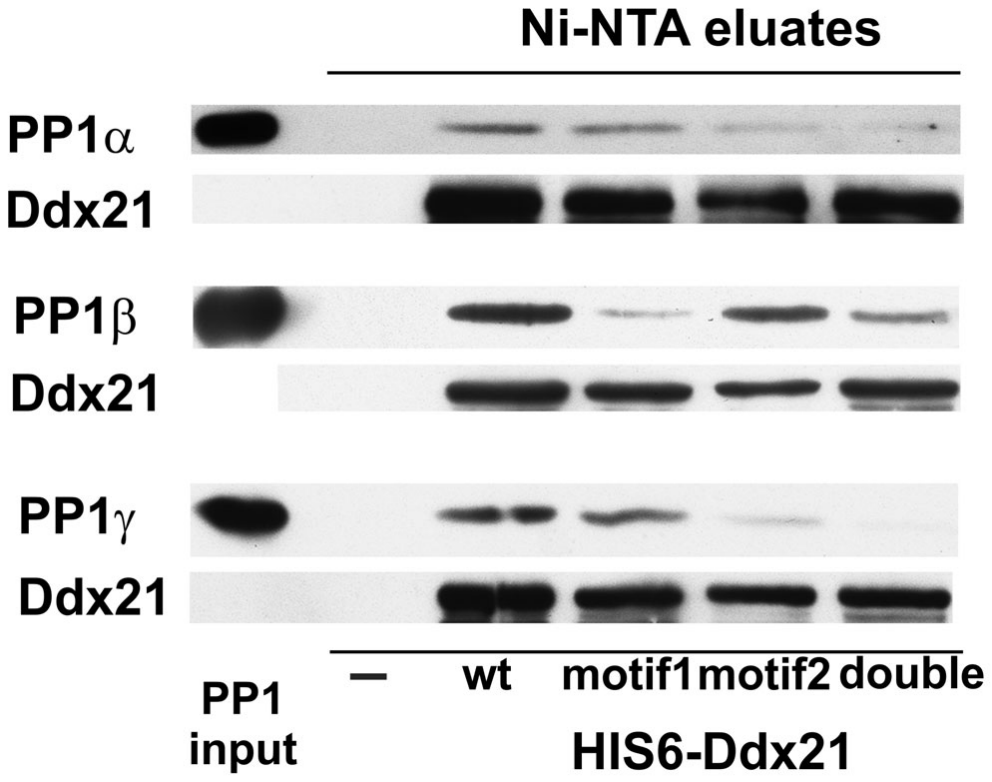
Pull down of purified, bacterially expressed PP1 isoforms via His-tagged RNA Helicase Ddx21. Bacterially expressed and purified PP1 (α, β, γ) and His6-Ddx21 (wt, motif1, motif2, double) were incubated together and then mixed with precleared Ni-NTA beads, all at 4°C. Each PP1 isoform was also incubated without Ddx21 (-) to verify unspecific binding of PP1 to the Ni-NTA beads. Enriched His6-Ddx21 and co-precipitated PP1 isoforms were washed and boiled in 2 X SDS-PAGE sample buffers. PP1 inputs, Ddx21 eluates and negative control (-) eluates were separated and analysed by western blot for the presence of Ddx21 and PP1.

Finally, we initiated *in vitro* pull downs with bacterially expressed and purified His6-Ddx21, incubated with PP1 isoforms (Figure 5) to support the findings in Figure 4. We incubated each isoform of PP1 (α, β, γ) either alone or with His6-Ddx21 (wt, mut1, mut2, double). After a pre-incubation, the Ddx21 proteins were enriched with Ni-NTA beads. Presence of Ddx21 and PP1, as co-eluant, was verified by the appropriate western blot which also showed no unspecific binding of PP1 to the Ni-NTA matrix (Fig. 5). Each PP1 isoform is enriched by His6-Ddx21_wt_ (Fig. 5, wt eluate lane), supporting the direct interaction of PP1 with the RNA helicase Ddx21. Interestingly, PP1α and PP1γ require the second RVxF motif within Ddx21 for their interaction with the helicase (Fig. 5 compare eluate lanes wt, mut1 with mut2, double), a need PP1β does not display (Fig. 5). The lack of PP1β interaction with Ddx21-His6_mut1_ is not maintained in Ddx21-His6_double_ (Fig. 5). This suggests that the observed interaction between Ddx21 (wt) and PP1β may rely on a larger number of secondary interactions, rather than on the canonical RVxF motifs alone.

### Towards the Identification of the Mitotic PP1-Ddx21 Target

The identification of this novel PP1 complex initiates the quest for the PP1-Ddx21 substrate(s). This could be Ddx21 itself, considering the helicase is a mitotic phospho-protein [40]. Alternatively, PP1-Ddx21 can target an unknown phospho-protein. In this case, the PP1-Ddx21 substrate may have already been identified as a Ddx21 interactor.

Both Prp8 and Ddx21 were previously identified by Lee and co-workers within one mitotic protein complex, the toposome [31]. This complex, named for the presence of the DNA topoisomerase TOPOIIα, further contains the pre-mRNA splicing factors Prp8 and hnRNP C1/2, the protein kinase SRPK1, the RNA helicases Dhx9/RHA and Ddx21/Gu and the structural protein SSRP1. The toposome complex stimulates the decatenation of condensed mitotic chromatin more efficiently than TOPOIIα alone [31], suggesting the other enzymes in this complex may play a supportive role.

**Figure 6:**
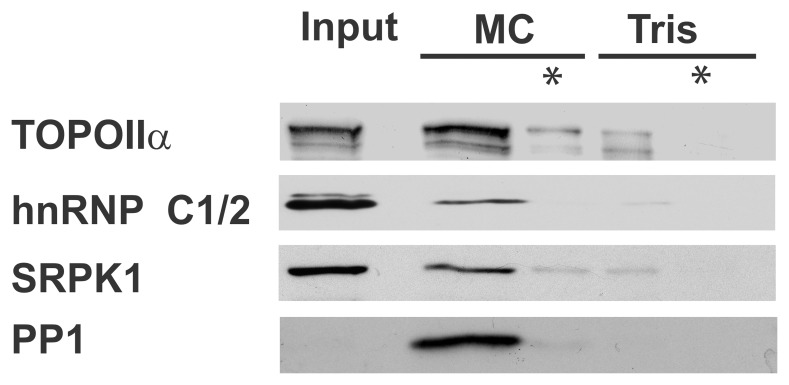
Toposome members as part of the mitotic phosphatome. Mitotic spindles were isolated (fraction 1-3) and MAPs and MTs separated as before (Fig. S1–2). Diluted MAPs (IN) were subjected to MC- or Tris-Sepharose affinity chromatography and proteins eluted with 1% SDS (MC, Tris respectively). Each eluate had a small precipitate after concentration which was loaded separately (*). Western blot analyses were performed with antibodies against indicated proteins.

**Figure 7:**
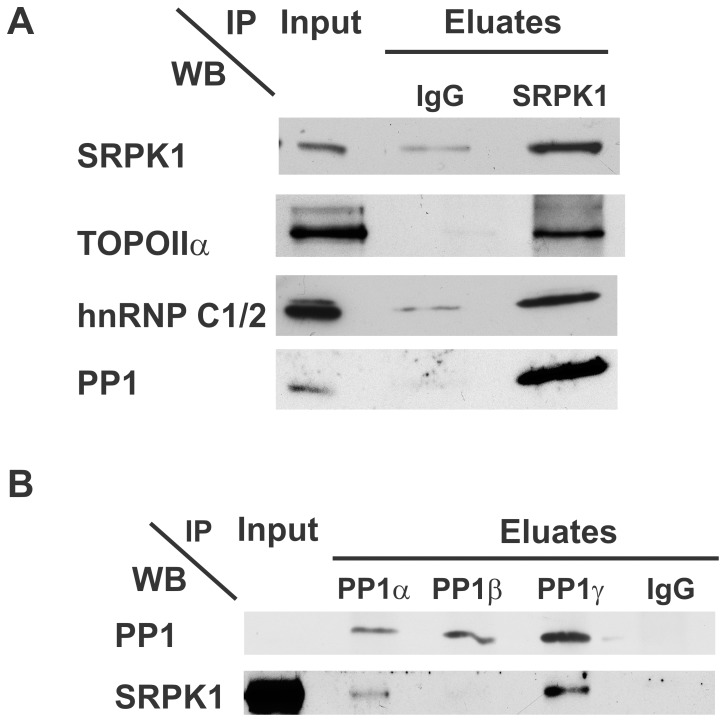
PP1 is a toposome interaction partner within the mitotic spindle proteome. A. Immunoprecipitation of SRPK1 identifies toposome components and PP1**.** Mitotic spindles were isolated and MAPs separated as in Fig. S1–2. Diluted MAPs (IN) were subjected to immunoprecipitations with SRPK1 or control (mouse IgG) antibodies. Bound proteins were eluted by boiling in 1X sample buffer. Western blot analyses were performed with antibodies against indicated proteins. **B.** PP1α, γ are the preferred interaction partners for SRPK1. Experiments were carried out as in **A**, except for the use of antibodies against human PP1 isoforms or control antibodies (PIS rabbit IgGs).

If PP1 interacts with the toposome during mitosis, we expect that the phosphatase interactor proteome, enriched from the mitotic spindle and chromatin associated proteome (fraction 3), will contain additional toposome components. Figure 6 clearly shows that apart from Prp8 and Ddx21 (Fig. 2, 3), this fraction indeed also enriches hnRNPC1/2, TOPOIIα, SRPK1 and SSRP1. Thus, Ddx21 could bring PP1 in contact with the mitotic toposome complex. This led us to investigate the toposome for potential interactors and/or substrates of the mitotic PP1-Ddx21 complex. We previously identified TOPOIIα as a nuclear PP1 interactor in HeLa cells, an interaction that can be released by RVxF-peptide displacement [11]. Mitotic spindle associated TOPOIIα is highly phosphorylated [31], [41] which makes it a potential target for serine/threonine phosphatases, prior to mitotic exit. Whether PP1-Ddx21 indeed dephosphorylates TOPOIIα *in vivo* during mitosis will require further studies. Other potential mitotic PP1-Ddx21 substrates are SRPK1-targeted phospho-proteins. The protein kinase SRPK1 phosphorylates Serine-Arginine (SR) repeats within the SR proteins, a family of key splicing factors, including ASF/SF2 [42]. ASF/SF2 plays a strategic role in interphase splicing but has recently also been implicated in mitosis by the mitocheck consortium [2]. The phosphorylation pattern of the SR proteins in general and ASF/SF2 in particular is complex, with various partial phosphorylation states each impacting splicing capacity. A recent publication showed that PP1 can counteract SRPK1 mediated phosphorylation of the splicing factor ASF/SF2 *in vitro* [42]. Furthermore, an independent study confirmed the direct interaction between PP1 and ASF/SF2 *in vivo* via the PP1-binding motif within the RNA binding domain of the splicing factor [43]. A third group identified ASF/SF2 and Ddx21 as co-immunoprecipitates of TIA-R, an RNA-binding protein with roles in RNA splicing and mRNA translation [44]. These observations suggest SRPK1, PP1, ASF/SF2, and Ddx21 to be functioning towards common goals. However, where Ddx21 is present at the mitotic perichromatin region [45], ASF/SF2 is released from chromosomes at mitotic onset and only returns to the chromatin at mitotic exit [46]. As such, ASF/SF2 is an unlikely target for the SRPK1 molecules present in the mitotic spindle and chromatin associated proteome. SRPK1 and PP1 may nonetheless target communal substrates, possibly within the toposome complex, since this contains active SRPK1 [31]. To verify their joint presence, we immuno-precipitated SRPK1 from the mitotic spindle and chromatin associated proteome of HeLa cells and probed for the presence of PP1. Our western blot analyses show that PP1 co-precipitates with the SR protein kinase, as does TOPOIIα and hnRNPC1/2 (Fig. 7A). To validate these results, we also performed a reciprocal immuno-precipitation with isoform specific PP1 antibodies. We identified SRPK1 as an interaction partner for PP1α and PP1γ but not PP1β (Fig. 7B). Overall, these data suggest that SRPK1 and PP1 can be part of a mitotic complex and have the capacity to regulate common mitotic substrates, other than ASF/SF2. Whether this target would be Ddx21 itself, a splicing factor within the toposome or an as yet unidentified substrate awaits our investigation.

## Discussion

Mitosis lasts approximately 3% of the average duration of a metazoan cell cycle, which makes the identification of mitotic protein complexes a difficult process. However, to understand cell growth and division and possibly pinpoint complexes with pharmaceutical potential, the identification of mitotic phosphoprotein phosphatase (PPP) complexes is key. Here we composed a strategy for the enrichment and identification of these complexes, based on previously published elements. We synchronize and harvest mitotic cells from which we isolate the mitotic spindle proteome (Fig. 1 and 1) together with centrosomal, centromere/kinetochore and chromatin associated proteins, similar to [32]. We then separate the microtubules (MT) and centrosome from their associated proteins (MAPs and centromere/kinetochore proteins), based on the largely salt-insoluble properties of the centrosome [47], [48]. Note that chromosome associated proteins such as helicases or Topoisomerase IIα are mainly soluble under such ionic strength. This step also reduces nonspecific binding via tubulin in downstream applications (Fig. S2). Finally, we enrich the PPP complexes from the soluble protein fraction (3a) via affinity chromatography (Fig. 2). Our approach is of general interest for researchers studying metazoan mitosis because the affinity chromatography step can be altered (e.g. from MC-Sepharose to e.g. α-Ddx21 or α-SRPK1 immunoprecipitations). This procedure is also applicable to various adherent metazoan cells as HEK293 cells synchronized with similar success and showed adequate PPP enrichment.

This method delivers novel insights on two levels. We show that a fraction of all PPPs, with the exception of PP5, is present within the mitotic spindle and chromatin associated proteome (Fig. 1B, fraction 3) and subsequently in the soluble fraction (fraction 3a) (PP1 Fig. 2C, 3, 5–6; PP2A, PP4, PP6 data not shown). Their presence reinforces their potential as mitotic regulators and encourages investigating PPP interactors in the mitotic spindle and chromatin associated proteome. For example, the clear enrichment of TIP41-like (TIPRL) (Fig. 1B, fraction 3) may offer inroads towards a functional mitotic annotation for this thus far elusive PPP interactor.

Second, the PP1 interactome is predicted to contain more than 200 candidates [13], some of which form stable and abundant complexes, repeatedly identified by high-throughput, quantitative studies. Others are under tight spatial-temporal control and will only be identified by a directed approach. This method is designed to isolate more transient and/or low abundance mitotic phosphatase complexes. Previous quantitative approaches using complete extracts from growing cells listed Ddx21 within the realm of low-abundance, at or below-threshold PP1 interactors (Fig. S4 and data not shown). Here, we showed that Ddx21 is a bona fide interactor, merely hidden underneath the most abundant complexes. Considering we only studied proteins remaining on the column after a medium-ionic strength wash (Fig. 2), manipulation of this approach could identify more mitotic PPP complexes.

Ddx21 is a DExD box superfamily 2 (SF2) RNA helicase which, based on sequence similarity [49], [50] and *in vitro* assays [51], [52], may help unwind dsRNA loops and fold ssRNA strands *in vivo*, essential events for RNA processing in growing cells. The precise regulation and function of Ddx21 during mitosis is particularly unclear as transcription is silenced and ribosome biogenesis considered inactive. In keeping with its proposed role in interphase, Ddx21 localizes in the nucleolar, dense fibrillar component (DFC) of unstressed cells [53]. Physicochemical stresses or down-regulation of the transcription factor c-Jun induce its fast relocation to the nucleoplasm [54], [55] as does expression of Ddx21-S171A [56], preventing the phosphorylation of S171 in growing cells [40]. Mutation of mitotically phosphorylated Ddx21 residues (S71A, S89A, S121A) [57], did not cause nucleoplasmic relocation of Ddx21 during interphase [56]. Thus, phosphorylation of Ddx21 fluctuates throughout the cell cycle and influences its localization and function [56]. Still, neither the kinases nor phosphatases responsible have been identified thus far. Here we present the first interaction of a phosphatase (PP1) with this RNA helicase *in vivo* and *in vitro* (Fig. 2, 3, 4, 5). Furthermore, PP1 and Ddx21 can co-localize in interphase nucleoli or at the mitotic perichromatin region [8], [45]. This brings further support for their interaction as does the positive influence of Ddx21 on PP1α activity towards a small molecule substrate (pNPP) (Fig. 4B). It is however well documented that phosphoprotein phosphatases as well as RNA helicases can be promiscuous enzymes in *in vitro* assays [10], [50]. Thus, the rise in PP1α activity in the presence of Ddx21 may not reflect an *in vivo* situation. Indeed, post-translational modifications of either partner and/or the presence of further complex components may alter their functional capacities. Moreover, we studied the interaction between PP1 and Ddx21 *in* *vivo* (Fig. 2, 3) and *in vitro* (Fig. 4, 5) and, not unexpectedly, identified slight variations in PP1 isoform preference, depending on experimental conditions.

The *in vivo* co-immunoprecipitations (Fig. 2, 3) did not discriminate between PP1 isoforms and further showed that peptide displacement was only partially successful in releasing Ddx21. Bacterially expressed Ddx21 requires motif 2 to interact with PP1α during overlay assays (Fig. 4C). We observed a similar preference during *in vitro* pull downs (Fig. 5). PP1β and PP1γ on the other hand showed different preferences for Ddx21, depending on the experimental conditions. A semi-denatured helicase, i.e. after SDS-PAGE separation and nitrocellulose transfer did not interact with PP1β nor PP1γ (Fig. 4C). However, when Ddx21 was directly incubated with PP1γ (*in vitro* pull down, Fig. 5C), motif 2 once again proved essential for interaction with the phosphatase. Under these conditions, PP1β weakly interacted with Ddx21, even when both motifs were dysfunctional. This suggested PP1β may rely on a series of secondary interactions with the helicase (Fig. 5).

In *in vitro* phosphatase assays, Ddx21 presence increased only PP1α activity (Fig. 4B). This did not exclude the potential interaction between PP1β, γ and Ddx21. Both motif 1 and 2 are conserved in the DDX21 homologs, found in Eukaryota from Protists to Animalia (data not shown). Motif 1 overlaps partially with one of the DEAD-box family defining helicase motifs [49]. Functional studies with various Ddx21 truncations identified a dsRNA unwinding domain, followed by an RNA folding domain at the C-terminal end [52]. Primary sequence analyses (Pfam, SMART), however, place the DExD-box specific helicase domain more N-terminal (aa205–532). Within the human Ddx21 sequence, both putative PP1 binding motifs locate within this helicase domain. It is therefore possible that binding of PP1 will hinder Ddx21 in its helicase function. Moreover, Ddx21 is one of the few helicases which has not been crystallized yet [58]. This may be due to the presence of the N-terminal low complexity region (aa1–200) or the absence of essential co-factors (e.g. nucleic acids/chromatin). The flexible structure of the helicase may also explain our observations regarding the interaction between PP1 isoforms and Ddx21 alleles in independent experimental set-ups. Indeed, the most pronounced difference between far-western assays (Fig. 4C) and *in vitro* pull downs (Fig. 5) is most likely the protein folding of Ddx21. This coincides with altered PP1 interaction capacities, particularly for PP1β and γ, suggesting that Ddx21 folding may be key for defining its preferred PP1 isoform *in* *vivo.*

Our results (Fig. 2, 5, 6) suggest that PP1 molecules can become part of the toposome during mitosis via their interaction with Ddx21. The toposome was isolated from G2/M derived extracts by Lee and co-workers [31], who also showed it aides TOPOIIα-mediated decatenation of chromatin. With the exception of Dhx9, we found all toposome members enriched in the mitotic spindle and chromatin associated phosphatome (Fig. 5). Moreover, we not only identified an interaction between PP1 and Ddx21 but also with other mitotic toposome members, i.e. the pre-mRNA splicing factor Prp8, and the Serine/Arginine Protein kinase SRPK1 (Fig. 6). These observations are supported by independent localization data, placing Ddx21 and TOPOIIα at the mitotic perichromatin region [45], similar to PP1 [17]. In interphase, many toposome members (TOPOIIα, Ddx21, Prp8, hnRNPC1/2) and PP1 isoforms are nuclear proteins although they maintain the capacity to interact with exogenous microtubules [59]. Thus, their inherent affinity for tubulin and the dismantling of the nuclear envelope at mitotic onset may be sufficient to bring these proteins towards the mitotic spindle. SRPK1 on the other hand is only partially nuclear in growing cells and does not interact with nuclear PP1 (our unpublished observations) nor with any of the toposome members [31]. SRPK1 accumulates in the nucleus only under stress conditions and at the onset of mitosis [60]. This suggests SRPK1 may be kept separate from the mitotic toposome and PP1 until mitotic onset (Fig. 6). Once in mitosis, they could form a complex which contains a protein kinase (SRPK1) and protein phosphatase (PP1) and multiple phospho-proteins [40], with potential SRPK1 motifs in at least TOPOIIα and SSRP1 (our observations). Thus, SRPK1 could help ensure the phosphorylation of the mitotic toposome members while PP1 would control their timely dephosphorylation. Apart from TOPOIIα and SSRP1, another potential substrate for their regulated phosphorylation could be Prp8. We identified this highly conserved splicing factor as a potential mitotic PP1 interactor (Fig. 2, 3) while the mitotic arrests of *prp8*-mutants cells underscore the key role of Prp8 and the spliceosome during mitosis [2], [61]. Also, *prp8*-mutant growth defects in *S. cerevisiae* are suppressed by a mutated PP1 regulatory subunit (Reg1) [62], supporting a role for PP1 in yeast spliceosome regulation. It remains to be investigated whether SRPK1, PP1 and additional kinases and phosphatases control the phosphorylation pattern of these proteins but the general concept of PP1 and SRPK1 controlling phosphorylation and function of a splicing factor (ASF/SF2) has been shown before [63]. Follow-up studies will help to answer these questions and define the expanding mitotic role of PP1.

## Materials and Methods

Chemicals were obtained from VWR or Bioshop Canada, unless indicated.

### Cells, Culturing, Synchronization and Mitotic Spindle Proteome Isolation

Human adherent cells (HeLa, HEK293; ATCC) were grown according to [32]. Mid-confluent cells are subjected to a thymidine (2 mM, 17 h) – nocodazole (130 mM, 9 h) block with a 7 h release in between. The mitotic spindle (MT) and associated proteins (MAPs) and interacting proteins (see introduction) are isolated according to [32]. Briefly, rounded G2/M arrested cells are released from culture plates by mechanical shake-off, collected and re-suspended in fresh media to progress into mitosis in the presence of Paclitaxel (5.85 µM). Mitotic cells are harvested washed (PBS, 5.85 µM paclitaxel, 2 µg/ml latrunculin) and re-suspended in lysis-buffer (100 mM PIPES-KOH pH 6.9, 1 mM MgSO4, 2 mM EGTA, 0.5% NP40, 5.85 µM paclitaxel, 2 µg/ml latrunculin, 200 µg/ml DNAseI, 10 µg/ml RNAse A, 5 U/ml micrococcal nuclease, 20 U/ml benzonase, protease inhibitors). The suspension is incubated at 37°C for 15 min with regular mixing and spun down (700 g, 3 min, room temperature - RT) to separate soluble proteins (fraction 1) from the MT/MAPs and interacting proteins and remnants of the cytoskeleton (actin and intermediate filaments). The latter are removed by i) using wash buffer (1 mM PIPES-KOH pH 6.9, 5.85 µM paclitaxel, 1 mM PMSF) to clean tube walls without disturbing the pellet ii) re-suspending pellet in wash buffer. Centrifugation (1500 g, 3 min, RT) separates the soluble actin/cytoskeleton remnants (fraction 2) from the MT/MAPs and interacting proteins (fraction 3). Average cell equivalent after shake off was 3*10E7 cells, with 43.2 mg protein overall. After separation, fraction 1 contains approx. 75% of the total protein, fraction 2 approximately 5% and fraction 3 approx. 20% of all proteins. Tubulin is significantly enriched during this procedure, thereby influencing the overall protein of fraction 3. Comparison of fractions 1 −3 were done by loading equal protein amounts. For fraction 1 and 2 this was based on protein measurements (3 µg/lane) while the volume for fraction 3 was defined by empirical loading and comparison to fractions 1, 2 via colloidal stainings. The required volume of fraction 3 contained approx. 6.3 µg protein (inclusive a disproportionately large amount of tubulin).

### Separation of MAPs and Interacting Proteins from MTs

Fraction 3 was re-suspended in Buffer A (25 mM Tris pH 7.5, 0.1 mM EGTA, 0.1% β-ME, 1 mM benzamidine, 0.1 mM PMSF) with 600 mM NaCl at RT to disrupt interactions between tubulin dimers and associated proteins [64]. A high speed centrifugation (52000 g, 35 min, RT) pellets the mitotic spindle and insoluble interacting proteins (fraction 3b). The supernatant contains MAPs and additional interacting proteins (fraction 3a) and was diluted with buffer A to a final concentration of 420 m M NaCl, ready for phosphoprotein phosphatase (PPP) complex isolation.

### Isolation of PPP complexes from the Mitotic Spindle and Chromatin Interacting proteome via Microcystin-Sepharose

The isolation is described in [11]. Phosphatase complexes were eluted with 1% concentrated using Amicon filters (10 K MWCO) prior to separation on SDS-PAGE. Proteins were identified after tryptic digestion and liquid chromatography mass spectrometry (LC-MS/MS) analysis on an LTQ-orbitrap system as described previously [65].

### Immunoprecipitation of PP1 and PP1-interacting Proteins

Antibodies against specific proteins or pre-immune serum IgG were covalently coupled to Protein A-Sepharose (Invitrogen) with dimethylpimelimidate (Sigma). Protein extracts from growing HeLa cells or MAP and interacting proteins (4 mg input for each experiment) were precleared with Protein A-Sepharose (20 min, 4°C) prior to incubation with the respective matrices (end/end, 4°C, 2 h). Next, matrices were washed with 3× 20 column volumes (CV) in (TBS plus 300 mM NaCl, 0.5% NP40) and 1× 20 CV PBS for Ddx21 or with 2× 20 CV (TBS plus 0.1% Tween20, 0.1% Triton) and 3× 20 CV TBS for SRPK1. Bound proteins were eluted by boiling in 1× Laemmli sample buffer. For western blot analyses, we loaded 40 µg input and equal volume fractions throughout, with the exception of the IP lanes (20× volume fraction).

### Bacterial Expression and Purification of Ddx21

**T**he DDX21 locus was amplified from a human cDNA library with PfuII Ultra (Roche) and cloned into the pRSET-A vector (Invitrogen). Products were verified by DNA sequencing (University of Calgary). pRSET-A-DDX21, transformed in DH5α and BL21(DE3) cells, was grown (37°C, 225 rpm, 14 h LB-Amp) to inoculate 1L LB-Amp 0.5% glucose (w/v) to OD_600 nm_ of 0.05. Cells were grown to OD_600 nm_ of 1.0; induced with 0.4 mM IPTG (60 min, 37°C); harvested and shock-frozen (−80°C) until further use. Pellets (0.5 L) were resuspended in lysis buffer (50 mM Tris pH7.5, 100 mM NaCl, 0.5% NP40, 0.5 mM PMSF, 0.5 mM benzamidine, 4 µM leupeptin, 1.5 µM pepstatin) to 25 ml and lysed with a french press (Sim-Aminco) (2 runs at 83 MPa exit pressure) and debris pelleted by centrifugation (23000 g, 4°C, 25 min). The supernatant was incubated with 3 ml pre-equilibrated SP-Sepharose (90 min, 4°C, end/end). Matrix was batch-washed with 20 CV of lysis buffer with 0.2 M NaCl and proteins eluted with 3 CV lysis buffer with 0.8 M NaCl. Sample was diluted to 150 mM NaCl and 10 mM imidazole added prior to loading on a pre-equilibrated Ni-NTA column (1.5 ml) (90 min, 4°C, end/end). Matrix was washed (50 mM Tris pH7.5, 1 M NaCl, 30 mM imidazole, 0.05% Triton, 0.5 mM PMSF, 0.5 mM Benzamidine, 4 µM leupeptin, 1.5 µM pepstatin), bound proteins eluted (50 mM Tris pH7.5, 300 mM NaCl, 300 mM imidazole, 0.5 mM PMSF, 0.5 mM benzamidine, 4 µM leupeptin, 1.5 µM pepstatin) and concentrated (Amicon filters 30 MWCO). Purification of bacterially expressed PP1 isoforms was described in [66].

### Cloning of DDX21 with Modified PP1-binding Motifs

Human Ddx21 contains 2 potential PP1-binding motifs; motif1 (aa202–208) and motif2 (aa440–444). pRSET-A-DDX21 was used as template for site-directed mutagenesis (Stratagene) to alter the sequence at motif1 from -KGRGVTF- to -KGAGATF- resulting in the protein His6-Ddx21_motif1_ and motif 2 sequence from -RTIIF- to -RTAIA- (His6-Ddx21_motif2_). We obtained the double mutant (His6-Ddx21_double_) by sub-cloning the motif 1 surrounding sequence into the motif 2 plasmid. All constructs were verified by DNA sequencing and expressed proteins by western blot analysis with a α-Ddx21 antibody. Primer sequences are available upon request.

### Far-Western Blot Analyses

Bacterially expressed His6-Ddx21 proteins, i.e. wild type, motif 1, motif 2 and double (1 µg each) were separated by SDS-PAGE and transferred to nitrocellulose membranes, along with control lanes containing 1 µg Bovine Serum Albumin or 15 µg crude HeLa lysate (data not shown). Membranes were incubated in 20% milk (w/v in PBS) to prevent unspecific binding, followed by an overlay with each DIG-labelled PP1 isoform. DIG labelling of PP1 isoforms was done according to the instructions of the manufacturer (Roche). Excess DIG-PP1 was washed away and remaining DIG-PP1 recognized by the α-DIG-HRP antibody (Pierce).

### In vitro Pull Down Assays

PP1 isoforms (α, β, γ) and Ddx21 proteins were purified as described. Purified proteins (300 ng PP1 and 200 ng Ddx21) or PP1 alone was mixed into 200 µl binding buffer (25 mM Tris pH 7.5, 5% (v/v) glycerol, 150 mM NaCl, 0.5% (v/v) Igepal630 (previously NP-40), 10 mM imidazole). Proteins were allowed to interact at 4°C for 30 min after which the equivalent of 20 µl precleared Ni-NTA bead slurry was added and interactions allowed to proceed for an additional 60 min at 4°C end over end. Beads are washed with 3× 25 volumes wash buffer (25 mM Tris, 450 mM NaCl (γ: 500 mM NaCl), 0.5% Tween-20 (γ: 0.75% Tween-20), 5% glycerol). Proteins remaining on the beads were boiled in 2× sample buffer and complete eluates loaded onto SDS-PAGE. Eluates were compared to an equal amount of the input PP1. Separated proteins were transferred to nitrocellulose membranes and analysed by western blot with Ddx21 and PP1 antibodies.

### PP1 Activity Assays with pNPP (p-nitrophenyl Phosphate) Substrate

We studied the impact of Ddx21 on the activity of the 3 isoforms of human PP1, all expressed in *E. coli*. Assays are essentially as in [67], [68]. Purified PP1 (25 ng) was incubated in assay buffer with increasing amounts of Ddx21, up to a 16-fold molar excess at 37°C for 10 min (20 µl). The small molecule substrate para-nitrophenyl phosphate (pNPP) was added to a final concentration of 12 mM. Reactions (50 µl) were incubated at 30°C for 1 hr and quenched with 150 µl 0.5 M EDTA, immediately followed by A405 nm measurements. Data points are mean of 3 replicates with standard deviation (mean ± S.D. n = 3). PP1 activity in the absence of Ddx21 was set to 1 with other points set out in function thereof. Similar results were obtained in at least 3 biological replicates, with Ddx21 derived from 2 host strains (DH5α and BL21-DE3).

### Generation of PP1 Isoform Specific Polyclonal Antibodies

Peptides identical to the C-termini of human PP1 isoforms (generated by Denis McMaster, University of Calgary - sequences available on request) were used to immunize New Zealand White rabbits, performed as in [69]. Pre-immune IgGs and PP1-antibodies were affinity purified from the respective sera via column chromatography with either Protein A-Sepharose or PP1-peptides crosslinked to CH-Sepharose. Full procedure and controls can be found in [70].

### Antibody Sources

Following persons donated antibodies against the indicated proteins: University of Calgary: M. Walsh (Rock2); MD Nguyen (Tpx2); JB Rattner (Cdk1); SP Lees-Miller (PP4; PP5; PP6, alpha4/IGBP1, TIPRL, SAPS1-3); Ebba Kurz (TOPOIIα); Rockefeller University NY: MM Konarska (Prp8). Further antibodies were purchased: α-tubulin (Sigma T-9026); HDAC1 (Cell Signalling # 2062); PP2Ac alpha (BD Biosciences 610555); DDX21 (Aviva Systems Biology); α-DIG-HRP (Pierce); SRPK1 (BD Biosciences 611072); SSRP1 (Biolegend USA); hnRNP C1/2 (Immuquest Ltd).

## Supporting Information

Figure S1 **Experimental set-up for the isolation of the mitotic spindle proteome.** Human cells (HeLa, HEK293) were grown to mid-confluence, arrested in S-phase by addition of 2 mM thymidine (17 h), released in fresh media (7 h) and arrested at G2/M with 130 mM nocodazole (9 h). Rounded, G2/M arrested cells were harvested by mechanical shake-off and released into fresh media to progress into mitosis. At the highest level of metaphase (microscopic observations of DAPI-stained chromosomes – data not shown) cells were harvested in the presence of paclitaxel (5.85 µM) to maintain mitotic spindles. Cells were lysed; soluble proteins (1) collected by centrifugation and the pellet washed with a low ionic strength buffer to remove intermediate and actin filaments (2). This allows harvest of the mitotic spindles and associated proteins (3) for further applications.
(TIF)

Figure S2 **Separation of the mitotic spindle proteome into microtubules and associated proteins.** HeLa cells were synchronized and the mitotic spindle proteome isolated as in Fig. S1 whereby only one half of the cells was treated with paclitaxel, indicated with (+ or -) to prevent microtubule collapse into soluble tubulin (*). The mitotic spindle proteome (fraction 3) was separated into soluble microtubule associated proteins (MAPs) (fraction 3a) and pelletable microtubules (MT) (fraction 3b). In each case + or – paclitaxel, samples were made exactly the same volume to allow a direct comparison and 1/1000 of the total volume of each fraction separated by SDS-PAGE and visualized by colloidal stain.
(TIF)

Figure S3 **LC-MS/MS results from MC-Sepharose enriched PPP interaction partners.** Excised bands (Fig. 2– BAND A-D) were trypsin digested and peptides identified by mass spectrometry (ESI-TRAP). Identified proteins are indicated with their common and uniprotKB name and identified peptides highlighted in bold.
(TIF)

Figure S4 **PP1 interaction with RNA helicases in the nucle(ol)i of interphase cells. A.** MS-based identification of Ddx21 as PP1-interactor. Nucleoli were enriched from unsynchronized HeLa cells grown in SILAC media and stably expressing either EGFP-PP1α or EGFP alone. Proteins were extracted and incubated with GFP-binder matrices [38]. Matrices were washed, prior to mixing of equal volumes, elution and quantitative MS analyses. Identified Ddx21 peptides are highlighted on the amino acid sequence. **B.** Co-immunoprecipitation of PP1 with Ddx21. Proteins were extracted from nuclei enriched from unsynchronized HeLa cells and incubated with Ddx21 or Pre-Immune IgG antibodies, crosslinked to PrA-Sepharose matrices. Bound proteins were eluted, separated by SDS-PAGE and analysed by western blot analyses.
(TIF)
