# Supplementary figures and images for: Isolation of Human Mitotic Protein Phosphatase Complexes: Identification of a Complex between Protein Phosphatase 1 and the RNA Helicase Ddx21
Source: PLoS One. 2012 Jun 28;7(6):e39510. doi: 10.1371/journal.pone.0039510 (PMC3386289; doi:10.1371/journal.pone.0039510)

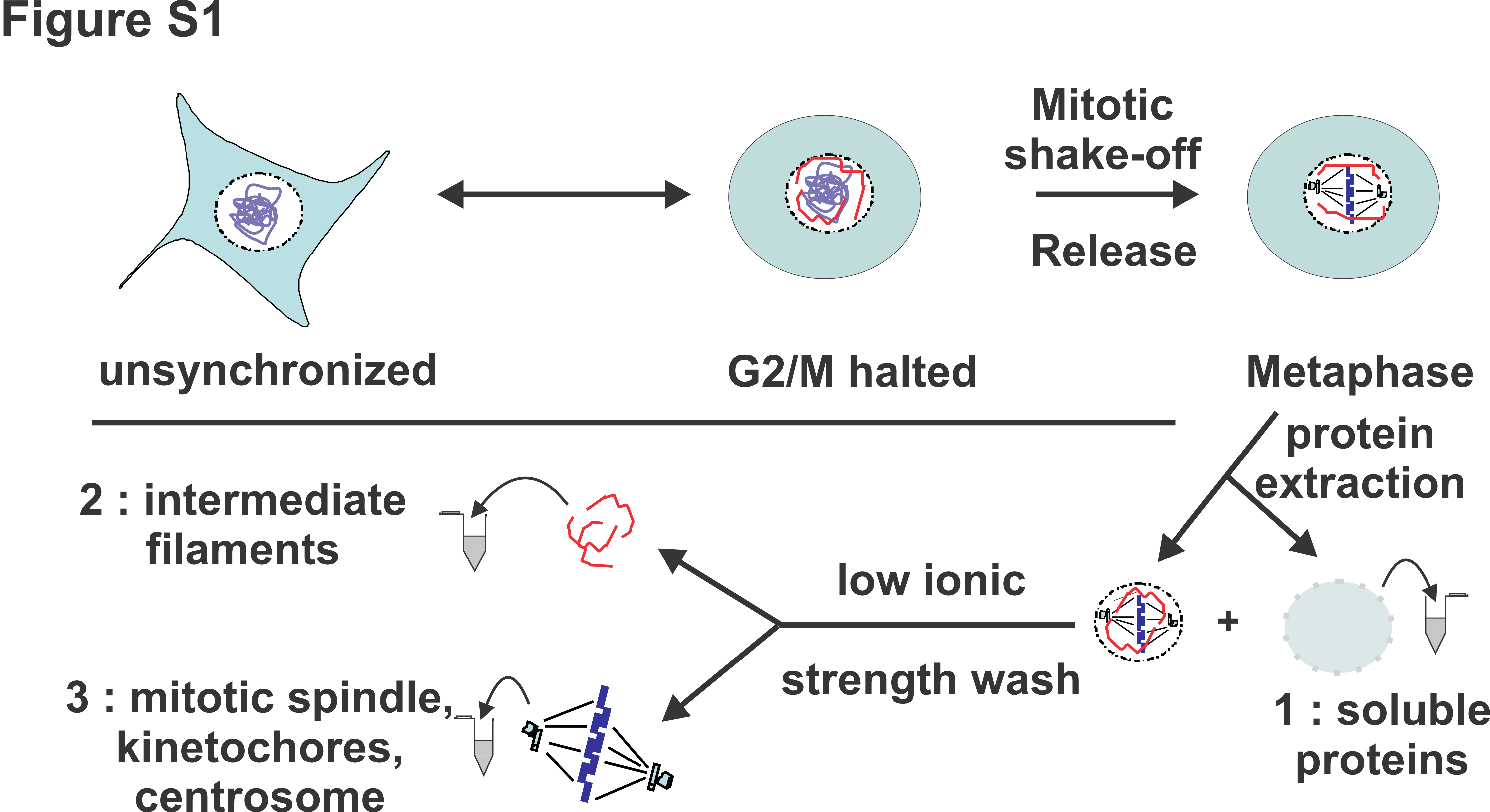

Supplement: Figure S1 — Experimental set-up for the isolation of the mitotic spindle proteome. Human cells (HeLa, HEK293) were grown to mid-confluence, arrested in S-phase by addition of 2 mM thymidine (17 h), released in fresh media (7 h) and arrested at G2/M with 130 mM nocodazole (9 h). Rounded, G2/M arrested cells were harvested by mechanical shake-off and released into fresh media to progress into mitosis. At the highest level of metaphase (microscopic observations of DAPI-stained chromosomes – data not shown) cells were harvested in the presence of paclitaxel (5.85 µM) to maintain mitotic spindles. Cells were lysed; soluble proteins (1) collected by centrifugation and the pellet washed with a low ionic strength buffer to remove intermediate and actin filaments (2). This allows harvest of the mitotic spindles and associated proteins (3) for further applications. (TIF) [file pone.0039510.s001.tif]

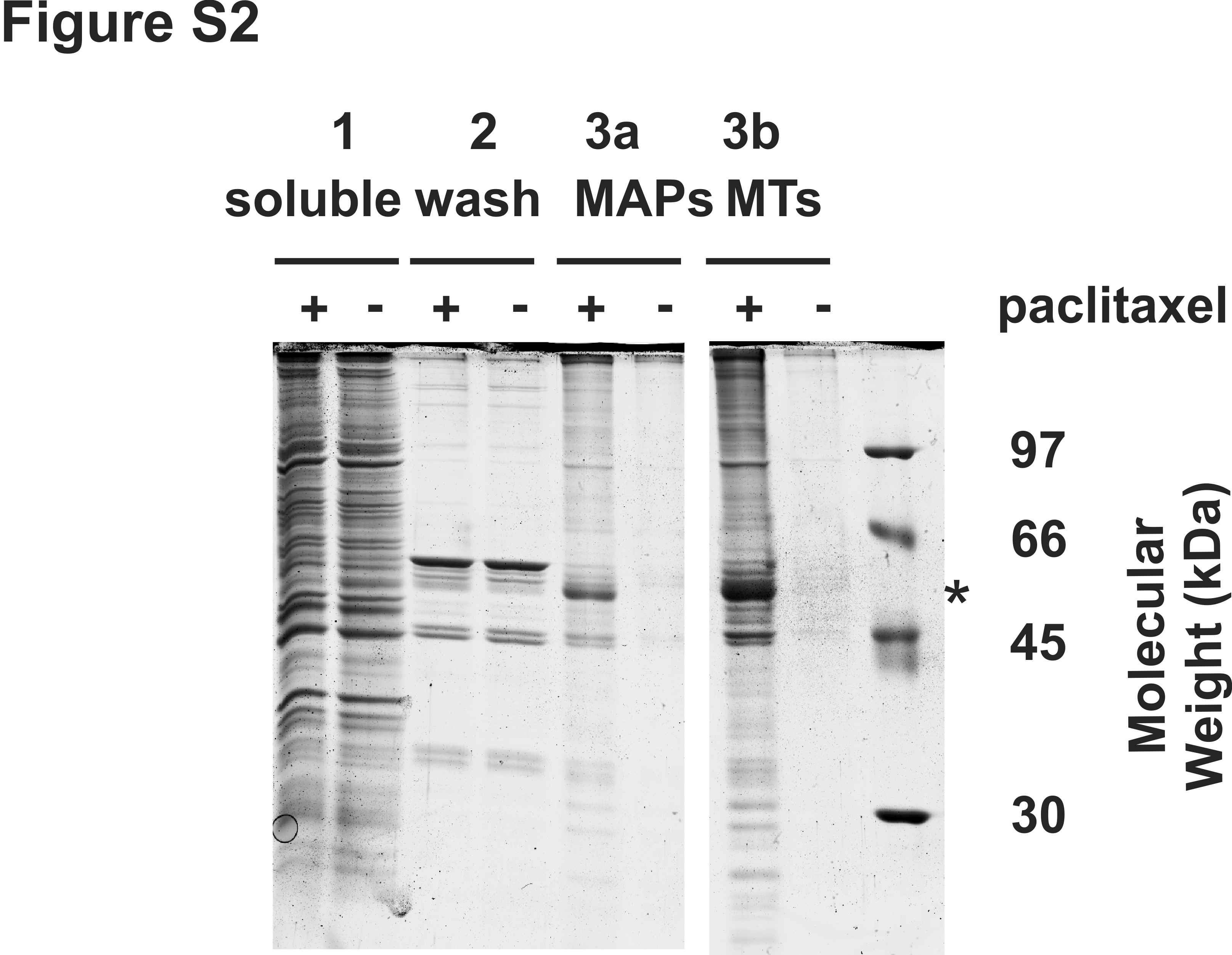

Supplement: Figure S2 — Separation of the mitotic spindle proteome into microtubules and associated proteins. HeLa cells were synchronized and the mitotic spindle proteome isolated as in Fig. S1 whereby only one half of the cells was treated with paclitaxel, indicated with (+ or -) to prevent microtubule collapse into soluble tubulin (*). The mitotic spindle proteome (fraction 3) was separated into soluble microtubule associated proteins (MAPs) (fraction 3a) and pelletable microtubules (MT) (fraction 3b). In each case + or – paclitaxel, samples were made exactly the same volume to allow a direct comparison and 1/1000 of the total volume of each fraction separated by SDS-PAGE and visualized by colloidal stain. (TIF) [file pone.0039510.s002.tif]

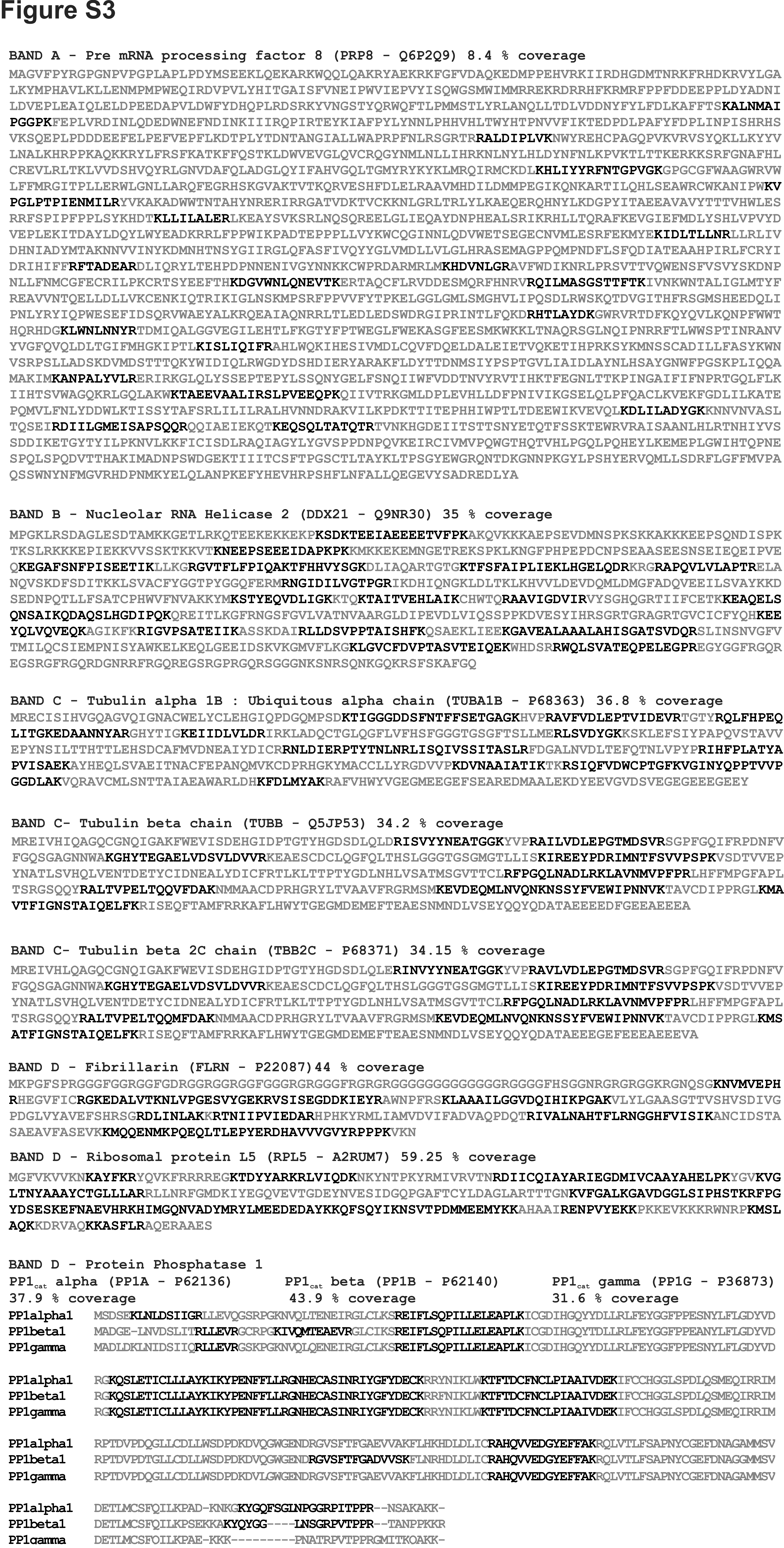

Supplement: Figure S3 — LC-MS/MS results from MC-Sepharose enriched PPP interaction partners. Excised bands (Fig. 2– BAND A-D) were trypsin digested and peptides identified by mass spectrometry (ESI-TRAP). Identified proteins are indicated with their common and uniprotKB name and identified peptides highlighted in bold. (TIF) [file pone.0039510.s003.tif]

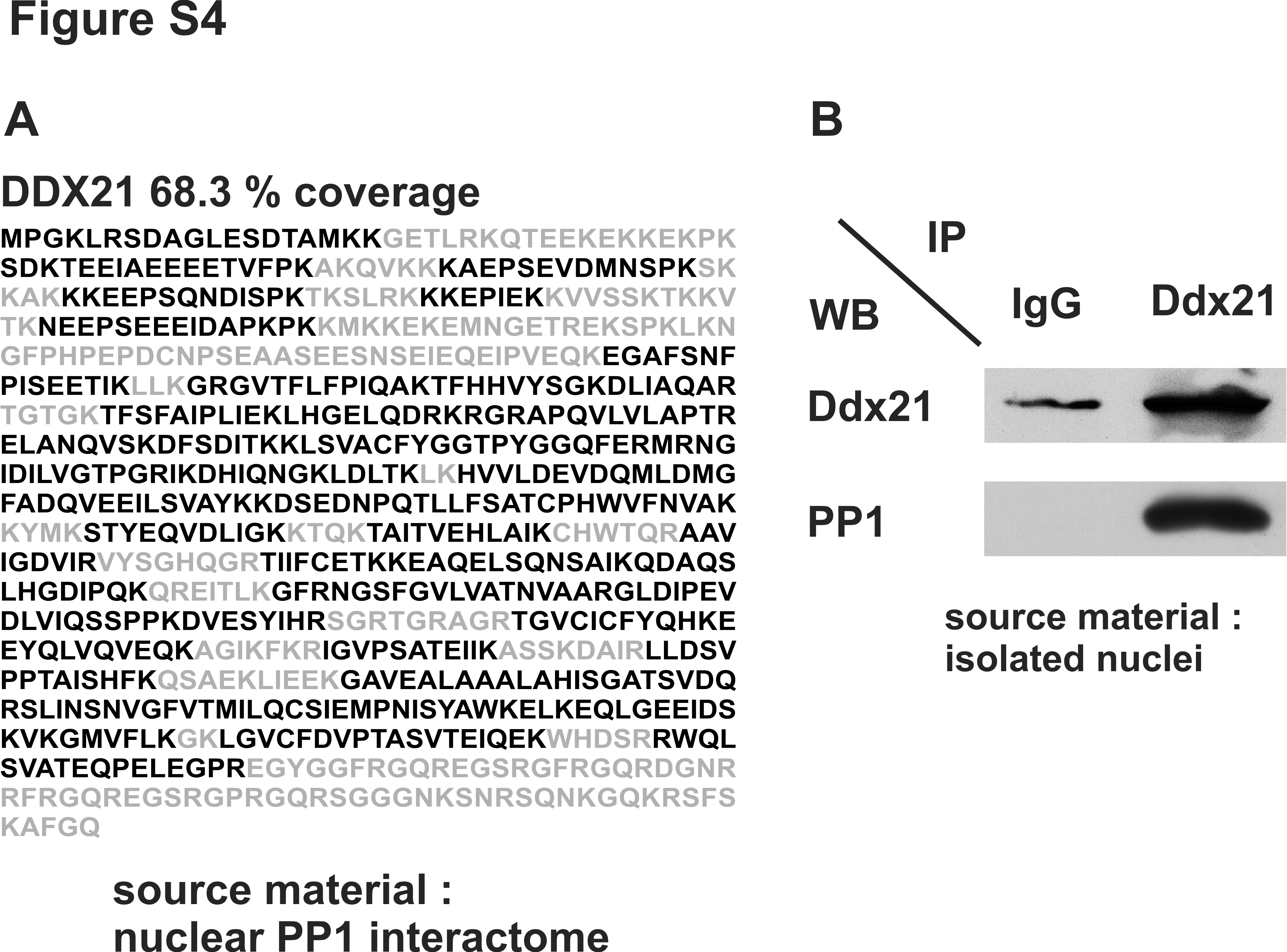

Supplement: Figure S4 — PP1 interaction with RNA helicases in the nucle(ol)i of interphase cells. A. MS-based identification of Ddx21 as PP1-interactor. Nucleoli were enriched from unsynchronized HeLa cells grown in SILAC media and stably expressing either EGFP-PP1α or EGFP alone. Proteins were extracted and incubated with GFP-binder matrices [38]. Matrices were washed, prior to mixing of equal volumes, elution and quantitative MS analyses. Identified Ddx21 peptides are highlighted on the amino acid sequence. B. Co-immunoprecipitation of PP1 with Ddx21. Proteins were extracted from nuclei enriched from unsynchronized HeLa cells and incubated with Ddx21 or Pre-Immune IgG antibodies, crosslinked to PrA-Sepharose matrices. Bound proteins were eluted, separated by SDS-PAGE and analysed by western blot analyses. (TIF) [file pone.0039510.s004.tif]
